# Development of humanized mouse and rat models with full-thickness human skin and autologous immune cells

**DOI:** 10.1038/s41598-020-71548-z

**Published:** 2020-09-03

**Authors:** Yash Agarwal, Cole Beatty, Sara Ho, Lance Thurlow, Antu Das, Samantha Kelly, Isabella Castronova, Rajeev Salunke, Shivkumar Biradar, Tseten Yeshi, Anthony Richardson, Moses Bility

**Affiliations:** 1 Department of Infectious Diseases and Microbiology, University of Pittsburgh, Pittsburgh, USA; 2 Department of Microbiology and Molecular Genetics, University of Pittsburgh, Pittsburgh, USA; 3 Hera Biolabs, Inc, Lexington, USA

**Keywords:** Animal disease models, Immunological models, Skin models, Regenerative medicine, Haematopoiesis

## Abstract

The human skin is a significant barrier for protection against pathogen transmission. Rodent models used to investigate human-specific pathogens that target the skin are generated by introducing human skin grafts to immunocompromised rodent strains. Infection-induced immunopathogenesis has been separately studied in humanized rodent models developed with human lymphoid tissue and hematopoietic stem cell transplants. Successful co-engraftment of human skin, autologous lymphoid tissues, and autologous immune cells in a rodent model has not yet been achieved, though it could provide a means of studying the human immune response to infection in the human skin. Here, we introduce the human Skin and Immune System (hSIS)-humanized NOD-*scid* IL2Rγ^null^ (NSG) mouse and Sprague–Dawley-Rag2^tm2hera^ Il2rγ^tm1hera^ (SRG) rat models, co-engrafted with human full-thickness fetal skin, autologous fetal lymphoid tissues, and autologous fetal liver-derived hematopoietic stem cells. hSIS-humanized rodents demonstrate the development of human full-thickness skin, along with autologous lymphoid tissues, and autologous immune cells. These models also support human skin infection following intradermal inoculation with community-associated methicillin-resistant *Staphylococcus aureus*. The co-engraftment of these human skin and immune system components into a single humanized rodent model could provide a platform for studying human skin infections.

## Introduction

The human skin provides the first line of host protection against environmental injury and host defense against pathogens^1, 2^. Several emerging pathogens, including community-associated methicillin-resistant *Staphylococcus aureus* (CA-MRSA), target the skin for infection and disease^1, 3–5^. Also, vector-borne infectious diseases such as Lyme disease and dengue fever are transmitted via inoculation into the skin by ticks and mosquitos, respectively^6^. Interactions between keratinocytes, skin fibroblasts, and cutaneous immune cells are involved in initiating the systemic immune response and abrogate pathogen replication and dissemination to other sites of replication^7–10^. Thus, the skin provides an ideal vaccination target for inducing immunity against various pathogens, as reflected by the development of several novel vaccine technologies directed at the skin, including skin-patch intradermal vaccines^11, 12^.

In vivo models for studying environmental insults and pathogens that target the skin and associated cutaneous immune cells primarily involve mice and rats^3^. These rodent models have improved mechanistic understanding of human diseases; however, significant differences exist between the skin and immune system of humans and rodents^3, 13^. Rodent skin microanatomy differs from human skin microanatomy due to the rodent skin lacking a multi-layered epidermis, eccrine and apocrine glands, and the papillary, reticular, and hypo-dermal regions of the dermal layer^14^. Human primary and secondary lymphoid tissues microanatomy differs significantly from that of rodent lymphoid tissues as well, with significant differences in red pulp to white pulp ratio in the spleen and lobulation of the thymus^15–17^. It is well-established that lymphoid tissue microenvironment, including stromal cells, plays a significant role in immune cell development^18^. Interactions between immune cells and stromal cells in non-lymphoid tissues, such as the skin, play a significant role in modulating tissue-associated immune responses^14^. Translational gaps may form between clinical studies performed with traditional rodent models, thus highlighting the need for humanized rodent models that can support the engraftment of both human skin and immune system components^1^.

To address the species gap between rodents and humans, researchers have engrafted the immunodeficient NOD-*scid* IL2Rγ^null^ (NSG) mouse model, which lacks mature lymphocytes and natural killer (NK) cells and possesses defects in innate immunity, with various human cells and tissues^19, 20^. Termed humanized-NSG mice, these models exhibit both human immune cell reconstitution and human lymphoid tissue growth and have been used to recapitulate clinical features of human diseases (including skin-associated diseases)^21–25^. Several separate reports demonstrate that immunodeficient mice support the engraftment of human skin^26, 27^. Adult human skin-derived from either medical wastes (elective plastic surgery)^28, 29^ or tissue culture-derived engineered skin (keratinocytes and fibroblasts)^24^ engrafts successfully in immunodeficient mice. Allogeneic adult human-peripheral blood mononuclear cells (PBMCs) have been introduced into these models to mimic human immune cell-skin interactions with infectious agents^24, 29^. Although these mouse models demonstrate successful engraftment and development of transplanted human skin and are amenable to the transplantation of allogeneic PBMCs, said platforms are not currently coupled with the engraftment of autologous lymphoid tissues that are critical for a de novo immune response. Humanized mouse models that combine human skin, human immune cells, and human lymphoid structures have yet to be established, despite their potential for developing a functional in vivo system, which could enable studies on human skin-immune cell interactions^30^. Humanized mouse models with human fetal-derived hematopoietic system and autologous lymphoid tissues are well-established^19, 20^. Additionally, full-thickness human fetal skin readily engrafts onto immunodeficient mice and develops into adult-like skin due to its high regenerative capability^31, 32^. Furthermore, human fetal skin exhibits low levels of major histocompatibility complex (MHC) I and II expression, which results in reduced immunogenicity compared to adult skin^31, 32^. Thus, human fetal-derived tissues and cells provide a feasible means to develop a humanized mouse model with autologous human skin and immune system.

Rats are a major model specie for long-term (greater than one year) studies^33^; thus, humanized rat models that combine human skin, human immune cells, and human lymphoid structures are being explored for use in long-term in vivo studies of human skin-immune interactions. Reports have demonstrated that nude rats (with moderate immunodeficiency) support adult human skin (split-thickness skin) engraftment and development, albeit host-mediated immune rejection occurs within a few months^34–36^. Evidence also suggests that nude rats support engraftment and development of full-thickness neonatal foreskin^37^. Recently, an immunodeficient rat, termed Sprague–Dawley-Rag2^tm2hera^ Il2rγ^tm1hera^ (SRG) rat, was developed to support engraftment of human cells and tissues into a larger and longer-life span rodent model; this model lacks mature T, B and NK cells^38, 39^. To date, humanized rat models co-engrafted with human full-thickness skin and autologous lymphoid tissues and immune cells (including cutaneous immune cells) remain to be developed and established^28, 40–42^.

Here, we utilize the immunodeficient NSG mouse and SRG rat models to generate rodent models with human skin, along with autologous lymphoid tissue(s) and autologous immune cells, termed human Skin and Immune System (hSIS)-NSG mice and SRG rats. Adolescent rodents received co-transplants of human full-thickness fetal skin, autologous lymphoid tissues, and autologous hematopoietic stem cells. Additionally, the human skin xenografts were inoculated with CA-MRSA to establish the susceptibility of these hSIS-humanized rodent models to human skin pathogens.

## Results

### The hSIS-humanized NSG mouse model supports the development of full-thickness human skin, autologous lymphoid tissues (thymus and spleen), and human immune cells

**Figure 1:**
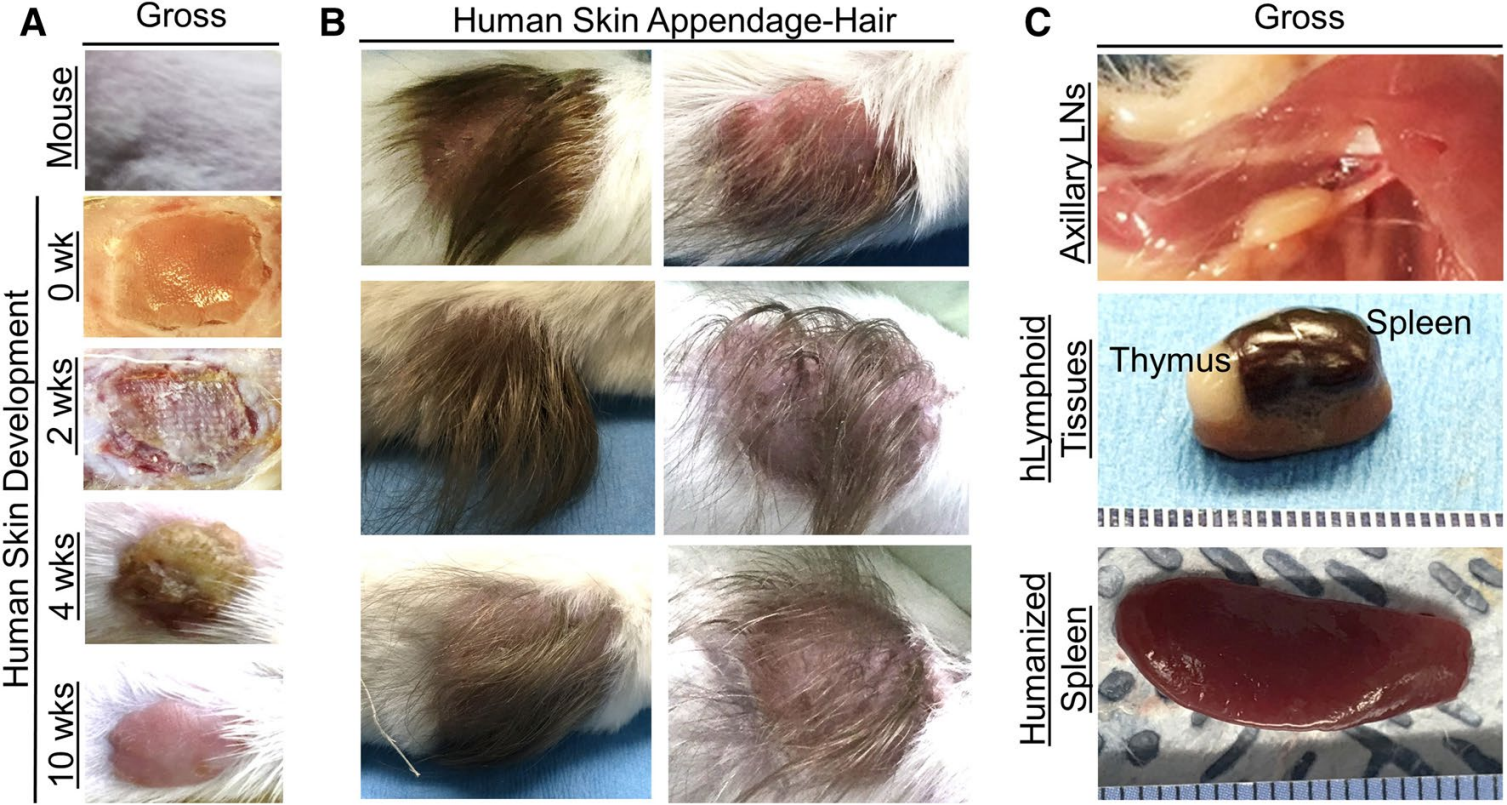
Development of human skin and lymphoid tissues in the human Skin and Immune System-humanized NSG mouse model. Transplantation of full-thickness human skin (derived from the scalp for skin with hair, or derived from dorsum for skin without hair) on the dorsum (**A**, **B**) and autologous lymphoid tissues in the kidney capsule (**C**) of NSG mice results in the engraftment and development of full-thickness human skin and lymphoid tissues. (**A**) Representative gross-photos at 0 (the day of transplantation), 2-, 4- and 10-weeks post-transplantation, with intact mouse skin as control (n = 4 per group). (**B**) Transplantation of full-thickness human skin from regions with significant hair follicles (scalp) in the human Skin and Immune System-humanized NSG mouse model results in the development of human hair, as exhibited in representative gross-photos at 12 weeks post-transplantation (n = 6). (**C**) Representative gross-photos of human lymphoid tissues (spleen and thymus tissues) and humanized lymphoid tissues (reconstituted-immunodeficient murine lymph node and spleen) at ten weeks post-transplantation (n = 4).

**Figure 2:**
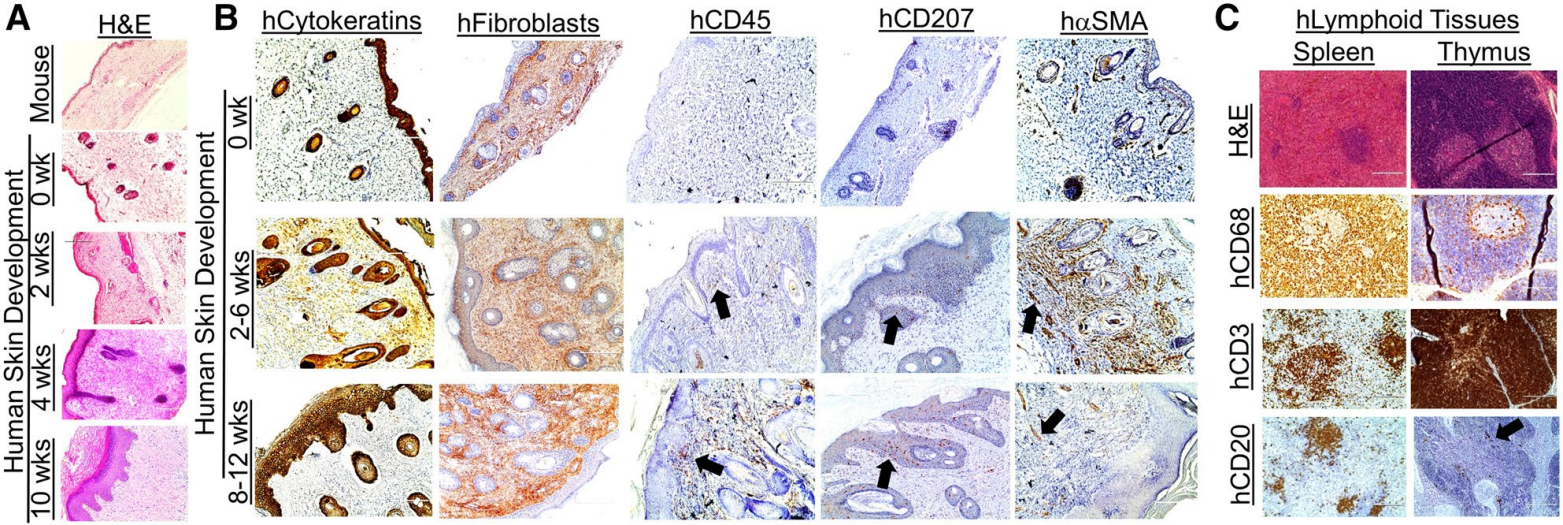
Development of human skin and immune cells in the human Skin and Immune System-humanized NSG mouse model. (**A**) Representative histological (H&E) analysis of the human skin in human Skin and Immune System-humanized NSG mice (n = 4) demonstrate the development of human adult-like skin, including the dermis, multicellular layer (> 5 layers) epidermis, and cornified envelope. Representative histological (H&E) analysis of the mouse skin demonstrate a thin-layer of epidermal cells, with a thin dermal layer. (**B**) Various human skin cells are present in the human skin, including keratinocytes (AE1/AE3+ cells, hCytokeratins+ cells), dermal fibroblasts (TE7+ cells, hFibroblast+ cells), cutaneous immune cells (hCD45+ cells), and Langerhans cells (hCD207+); alpha-smooth muscle actin-expressing blood vessel cells (hα-SMA+ cells) are present in the human skin xenograft and expand during wound healing and contract after healing (n = 4). The black arrows denote representative IHC+ cells. (**C**) Representative histological and immunohistochemical analysis of the human spleen and thymus (both under the kidney capsule) in human Skin and Immune System-humanized NSG mice demonstrate the development of those lymphoid tissues at ten weeks post-transplantation, with human macrophages (hCD68+), T cells (hCD3+), B cells (hCD20+) present in the tissues (n = 4) Scale bars: 200 μm.

**Figure 3:**
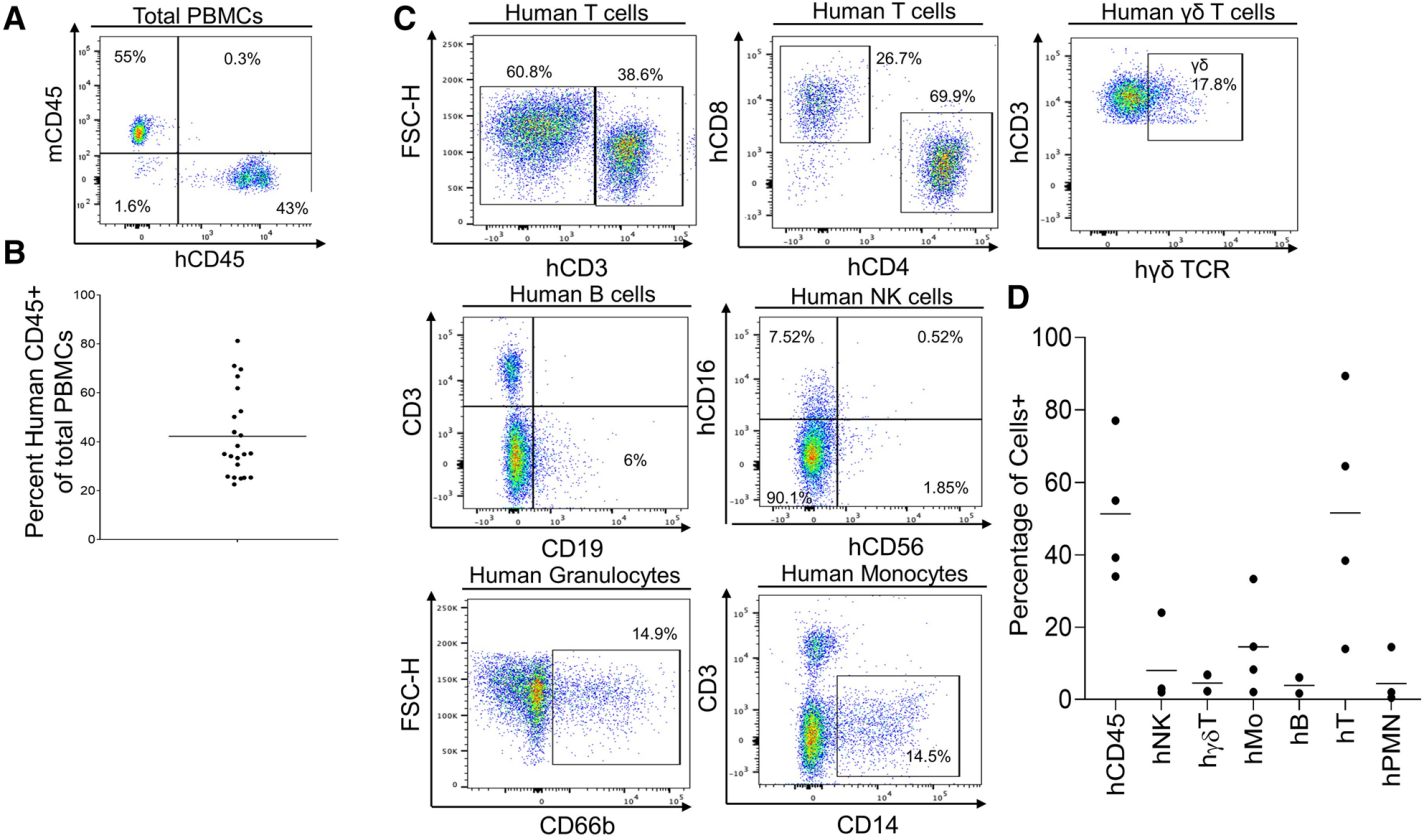
Development of human peripheral blood mononuclear cells in the human Skin and Immune System-humanized NSG mouse model. (**A**) Representative flow cytometry analysis of human immune cell (hCD45+) reconstitution in peripheral blood mononuclear cells (PBMCs) of human Skin and Immune System-humanized NSG mice at 10–12 weeks post-transplantation demonstrates high levels (> 10%) of human immune cells in the blood. (**B**) Quantification of human immune cell reconstitution (n = 22; 3 independent experiments) in PBMCs of hSIS-humanized mice at 10 weeks post-transplantation. (**C**) Representative flow cytometry analysis of human PBMCs (hCD45+ PBMCs) in human Skin and Immune System-humanized mice at 10–12 weeks post-transplantation demonstrates readily detectable levels of various human immune cell types (B cells-hCD19+hCD3- human PBMCs, αβ T cells-hCD3+ human PBMCs, hCD4+ T cells, hCD8+ T cells, hγδTCR+ T cells- hγδ TCR+ CD3+ human PBMCs, natural killer cells (NK)-hCD57+ hCD3- human PBMCs, monocytes (hMo)-hCD14+ CD3- human PBMCs, and granulocytes (hPMN)-hCD66b+ hCD3- human PBMCs). (**D**) Quantification of human immune cell subtypes (n = 4) in human PBMCs (hCD45+ PBMCs) of human Skin and Immune System-humanized mice at 10–12 weeks post-transplantation.

We previously demonstrated that NSG mice support the development of human lymphoid tissues (thymus and spleen) along with autologous immune cells following engraftment of fetal tissues and autologous hematopoietic stem cells^17^. Several reports demonstrated that immunodeficient mice support the development of human skin following engraftment of human fetal skin^43, 44^. Here, we hypothesize that NSG mice will support co-engraftment of human full-thickness fetal skin, autologous fetal lymphoid tissues, and autologous hematopoietic stem cells. Furthermore, we hypothesize that NSG mice will facilitate human skin and lymphoid tissue development, as well as enable systemic human immune cell reconstitution in transplanted human tissues and the blood. We processed human fetal spleen, thymus, and liver organs into ~ 1 mm^3^ pieces and isolated autologous human CD34^+^ hematopoietic stem cells from the fetal liver, then transplanted the tissues and hematopoietic stem cells into irradiated NSG mice. Human skin tissues were obtained from the scalp and dorsum of donors and were used in developing human skin engraftments with and without hair in the mouse model, respectively. Full-thickness human fetal skin was processed via removal of excess fat tissues attached to the subcutaneous layer of the skin, then engrafted over the rib cage, where the mouse skin was previously excised. In three cohorts, the overall success of the human immune system and autologous full-thickness skin development and maintenance for ten weeks or greater was over 75% (Supplementary Fig. 1). Gross analysis of the human skin xenograft in the hSIS-NSG mouse model beginning at two weeks post-transplantation demonstrates wound healing and maturation into adult-like human skin, which is evident at ten weeks post-transplantation (Fig. 1A, Supplementary Fig. 2). A limitation of the human skin in the hSIS-NSG mouse model is the development of dry skin (resulting in hardening) and early signs of murine hair loss (suggestive of graft-versus-host disease) at 20 weeks post-transplantation (Supplementary Fig. 2). The human skin in hSIS-NSG mice also develops human skin appendages, with human hair evident at 12 weeks post-transplantation (Fig. 1B). Gross analysis of human spleen and thymus xenografts in hSIS-humanized NSG mice at ten weeks post-transplantation demonstrated the growth of those lymphoid tissues under the kidney capsule (Fig. 1C)^17^. In addition to supporting the growth of human spleen and thymus tissues, the hSIS-humanized NSG mouse model supports the reconstitution of the immunodeficient-murine lymph nodes and spleen (Fig. 1C)^17^. Histochemical analysis of the human skin in hSIS-humanized NSG mice demonstrates the development of the human skin xenograft; the microanatomy of the human skin at ten weeks post-transplantation is comparable to adult human skin, with multiple layers of cells present in the epidermis (Fig. 2A, Supplementary Fig. 3). The human skin xenograft exhibited multiple layers of human keratinocytes (AE1/AE3, pan-cytokeratin antibody+ cells) in the epidermis and dermal fibroblasts (Anti-Fibroblasts Antibody+ cells) in the dermis (Fig. 2B). Additionally, the human skin exhibited human immune cell repopulation (human CD45^+^ cells), including Langerhans cells (hCD207+ cells), macrophages (hCD68+), and T cells (hCD3+) (Fig. 2B, Supplementary Figs. 4 and 5). The human skin in hSIS-humanized NSG mice also exhibited upregulation of alpha-smooth muscle actin-positive (α-SMA^+^) cells (i.e., blood vessel cells^45^) during revascularization and wound healing (~ 2 weeks post-transplantation), followed by a reduction in α-SMA^+^ cells in the healed skin (~ 10 weeks post-transplantation) (Fig. 2B). Histochemical analysis demonstrates the development of the co-transplanted human lymphoid tissues (spleen and thymus) in the renal capsule (~ 10 weeks post-transplantation) (Fig. 2C)^17^. Human thymus tissue in hSIS-humanized NSG mice exhibits T-cell (human CD3^+^ cells) reconstitution, with few B cells in the tissue (Fig. 2C)^17, 46, 47^. Macrophage reconstitution (human CD68^+^ cells) in the human thymus tissue is restricted to the medulla (Fig. 2C)^17, 46, 47^. Human spleen tissue in hSIS-humanized NSG mice exhibits macrophage reconstitution, with macrophages predominately in the red-pulp (Fig. 2C)^15, 16^. The human spleen tissue in hSIS-humanized NSG mice exhibits T and B cell repopulation (human CD3^+^ and CD20^+^ cells), with lymphocytes predominately in the white-pulp (Fig. 2C)^15^. Analysis of the PBMCs in hSIS-humanized NSG mice showed human immune cell reconstitution (hCD45+ cells) (Fig. 3A,B). Various human immune cell (hCD45+) subtypes, namely, αβ T cells (hαβ T cells with a CD4:CD8 ratio average of 1.34:1), γδ T cells (γδ T), NK cells (hNK), B cells (hB), monocytes (hMo) and granulocytes/polymorphonuclear neutrophils (hPMN) were reconstituted in the peripheral blood of the hSIS-humanized NSG mouse model (Fig. 3C,D), with analysis performed using appropriate flow cytometry assay controls (Supplementary Fig. 6).

**Figure 4:**
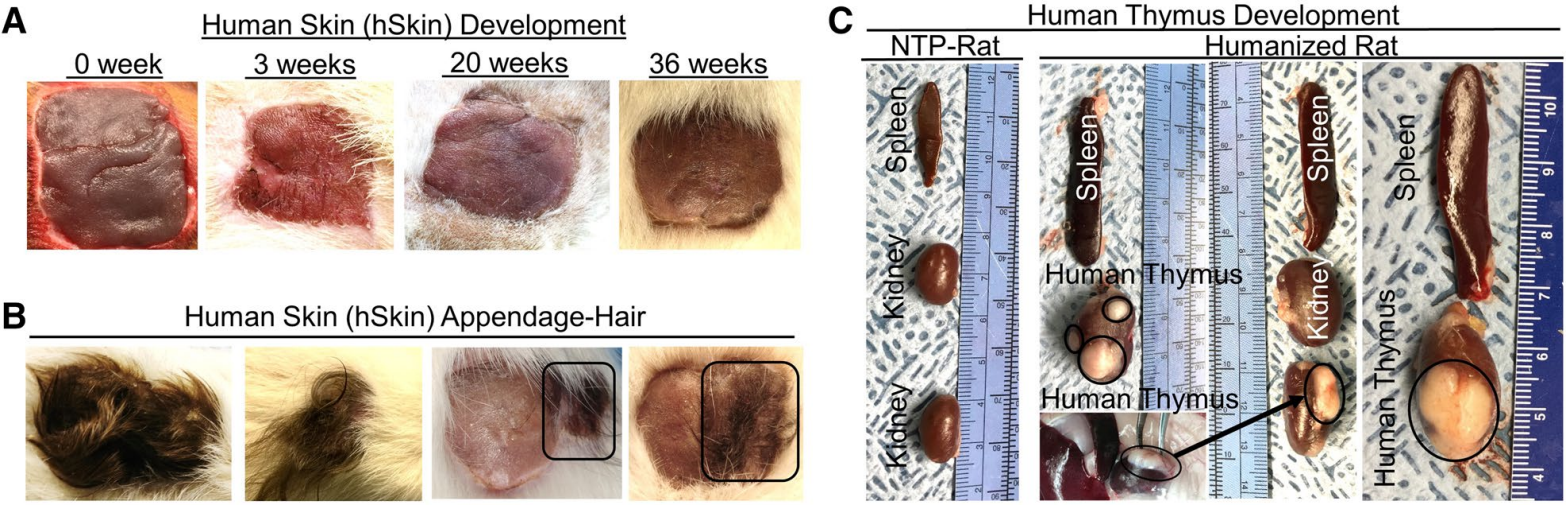
Development of human skin and lymphoid tissues in the human Skin and Immune System-humanized SRG rat model. Transplantation of full-thickness human fetal skin on the dorsum (**A**, **B**) and autologous fetal lymphoid tissues (thymus and liver) in the kidney capsule (**C**) of SRG rats results in engraftment and development of full-thickness human skin and primary lymphoid tissue (thymus) (n = 4). (**A**) Representative gross-photos at 0- (the day of transplantation), 3-, 20-, and 36-weeks post-transplantation demonstrate human fetal skin engraftment and development (using donor skin obtained from the dorsum). (**B**) Transplantation of full-thickness human fetal skin, derived from regions with significant hair follicles (scalp), on SRG rats with (left panel) (n = 2) or without (right panel) (n = 2) co-transplantation of thymus and hematopoietic stem cells results in the development of human hair as exhibited in representative gross-photos at six months post-transplantation. In the right panel, autologous human skin from dorsum and scalp were co-transplanted to demonstrate human hair only grows in human skin tissue with preexisting hair follicles (scalp; identified with black box). (**C**) Representative gross-photos of lymphoid tissues (human thymus in the kidney capsule and rat spleen) at nine months post-transplantation demonstrates the development of lymphoid tissues compared to non-transplanted SRG rat (n = 4). The black circles denote human thymus tissues.

**Figure 5:**
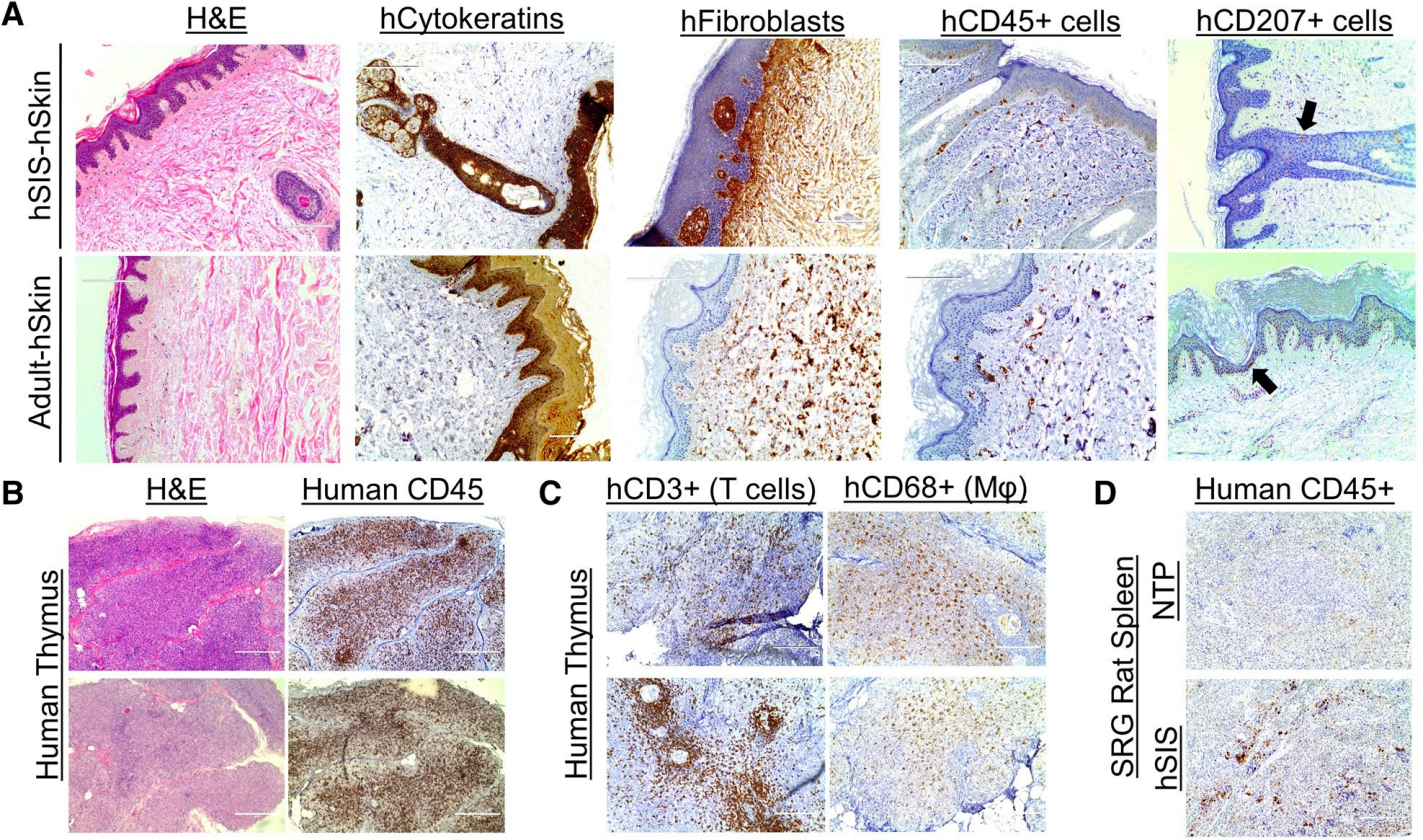
Development of human skin and immune cells in the human Skin and Immune System-humanized SRG rat model. Representative immunohistochemical analysis of the human skin in the human Skin and Immune System-humanized rat (n = 4) demonstrates the development of human skin, including dermis, multi-layered (> 5 layers) epidermis, and cornified envelope, which are hallmarks of adult-human skin (Adult-hSkin) (n = 2) (**A**). Various human skin cells are present in the human skin, including keratinocytes (AE1/AE3+ cells, hCytokeratins+ cells), dermal fibroblasts (TE7+ cells, hFibroblast+ cells), cutaneous immune cells (hCD45+ cells), and Langerhans cells (hCD207+; black arrows denote IHC+ cells) (n = 4). (**B**) Representative histological and immunohistochemical analysis of the human thymus (under the kidney capsule) in the human Skin and Immune System-humanized SRG rat demonstrates the development of human thymus tissue at nine months post-transplantation, with human immune cells (human CD45+), including (**C**) high levels of T cells (hCD3+) and macrophages (hCD68+) (n = 4). (**D**) The rat spleen in the human Skin and Immune System-humanized SRG rat model (n = 4) is also reconstituted with human immune cells (Humans CD45+); non-transplanted (NTP) SRG rat (n = 2) was used as a staining control. Scale bars: 200 μm.

**Figure 6:**
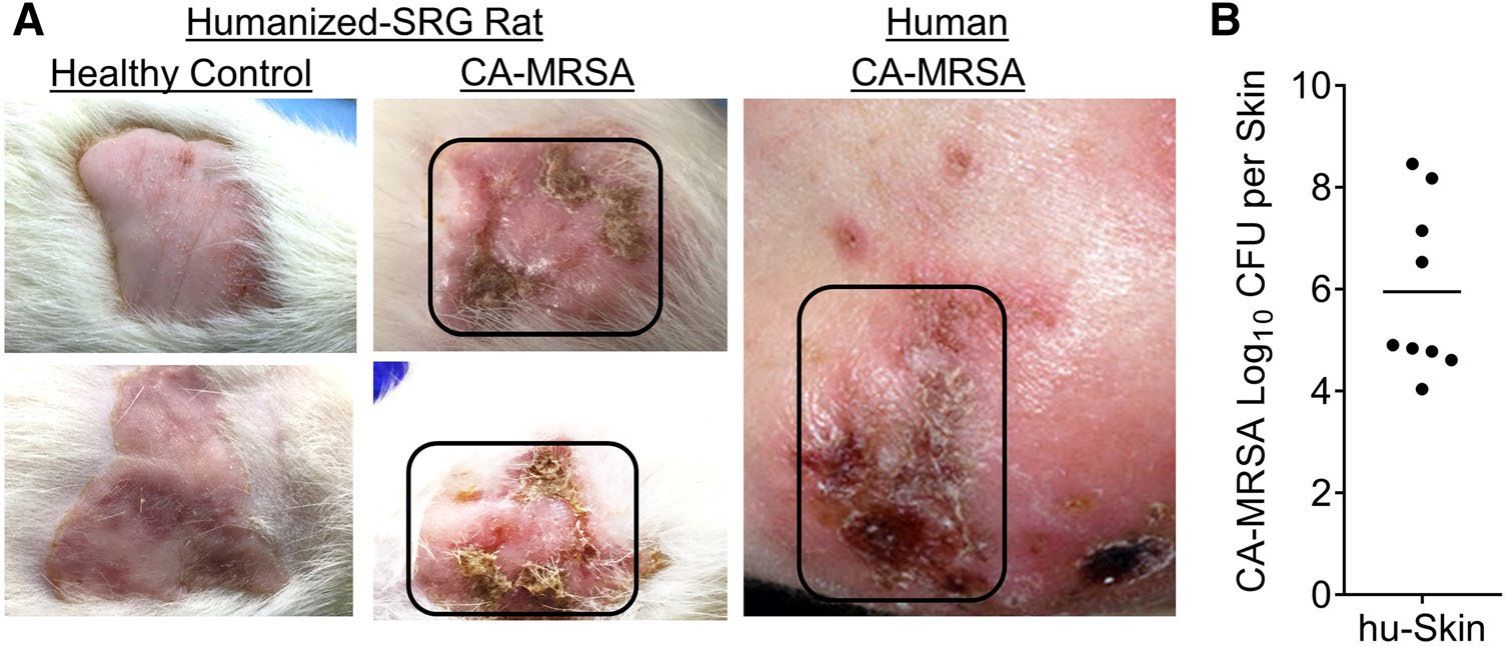
Human skin xenograft on SRG rats supports CA-MRSA infection. Community-associated methicillin-resistant *Staphylococcus aureus* (CA-MRSA) was inoculated into human skin xenograft (intradermal injection) on humanized (human skin) SRG rats (n = 9) to demonstrate that the human skin xenograft supports CA-MRSA infection. (**A**) Gross analysis of the skin tissues were examined in CA-MRSA inoculated humanized (human skin) SRG rats (n = 9) and compared to CA-MRSA infected human skin (Patient CA-MRSA skin photo credit: S. Camazine); healthy control-human skin in humanized (human skin) SRG rats (n = 5) were also used as controls to determine skin lesions. (**C**) The skin lesions in the CA-MRSA inoculated humanized (human skin) SRG rats (n = 9) supports high CA-MRSA bacteria load as measured at six weeks post-infection.

### The hSIS-humanized SRG rat model supports the development of full-thickness human skin, thymus tissues, and human immune cells

Although humanized mice have provided in vivo platforms for modeling human diseases, the short-life span and small tissue size/volume of mice are a major limitation for long-termed and preclinical studies, respectively. We hypothesized that a larger immunodeficient rodent model, with a longer life span, namely rat, would support the development of an in vivo model for long-term studies (> 6 months) and provide large tissue volume/size. We further hypothesized that the co-transplantation of human full-thickness fetal skin, autologous fetal thymus tissues, autologous fetal liver tissues, and autologous fetal-liver derived hematopoietic stem cells into SRG rat would enable the development of a model containing human skin, autologous thymus, and autologous human immune cells, termed hSIS-humanized SRG rat. Human fetal thymus and liver tissues (~ 1 mm^3^ pieces) were implanted into the kidney capsules of irradiated SRG rats, and those rats were immediately transplanted using autologous hematopoietic stem cells. Excess fat tissue was removed from the human fetal skin, and the tissue was subsequently engrafted over the rib cage, where the rat skin was previously excised. SRG rats were transplanted using human skin plus CD34+ human hematopoietic stem cells and thymus tissue, or with human skin only; 100% of the rats successfully engrafted and developed the transplanted human tissues (Supplementary Fig. 7). However, a human immune cell reconstitution of the blood in the transplanted rats was not observed in any group using this transplantation methodology (data not shown). Gross analysis of the human skin in rats transplanted with human CD34+ human hematopoietic stem cells plus thymus and skin demonstrated wound healing, beginning at three weeks post-transplantation, and maturation into adult-like human skin over time (Fig. 4A). The human skin transplanted onto SRG (with or without lymphoid tissue plus CD34+ human hematopoietic stem cells) also support the development of human skin appendages (hair) (Fig. 4B). Gross analysis of the lymphoid tissues in hSIS-humanized SRG rats at nine months post-transplantation showed the growth of the human thymic tissue in the kidney capsule and marginal increase in the size of the rat spleen (Fig. 4C). The human skin in hSIS-humanized SRG rats exhibited development of multi-layered human keratinocytes (AE1/AE3, pan-cytokeratin antibody+ cells) in the epidermis, and dermal fibroblasts (Anti-Fibroblasts Antibody+ cells) in the dermis, both of which are comparable to adult human skin (Adult-hSkin) and differ from rat skin (Fig. 5A, Supplementary Fig. 8). The human skin in hSIS-humanized SRG rats exhibited a reconstitution of human immune cells (hCD45^+^ cells), including Langerhans cells (hCD207^+^ cells), comparable to adult human skin (Adult-hSkin) (Fig. 5A). Histochemical analysis of human thymus tissue in hSIS-humanized SRG rats at nine months post-transplantation demonstrates thymus development, including the presence of thymic lobules (Fig. 5B). Human thymus tissues in the hSIS-humanized SRG rat exhibits human immune cell (human CD45^+^ cells) reconstitution, including T cell (human CD3^+^ cells) and macrophage (human CD68^+^ cells) reconstitution (Fig. 5B,C). Additionally, human thymic T cells exhibit cytokine response to stimulation using CD3/CD28 beads (Supplementary Fig. 9). Human immune cells in the hSIS-humanized SRG rat also reconstitute the immunodeficient-rat spleen (Fig. 5D).

### The human skin xenograft in immunodeficient rodent models supports community-associated methicillin-resistant *Staphylococcus aureus* infection

Community-associated methicillin-resistant *Staphylococcus aureus* (CA-MRSA) infection represents a significant public health threat ^5^; thus, in vivo models that enable investigation of human skin infections are of significance. In order to demonstrate that human fetal skin-derived skin xenografts in immunodeficient rodents provide a means for investigating human skin infections, we inoculated (intradermal) the adult-like human skin xenografts with CA-MRSA USA300. We observed lesions in the human skin in SRG rats inoculated with CA-MRSA, which were comparable to lesions in CA-MRSA patients; those human skin-lesions in the rats exhibited CA-MRSA bacterial growth (Fig. 6). Inoculation of CA-MRSA in the human skin xenograft in the NSG mouse model at 10–12 weeks post-transplantation, also supports bacterial growth (Supplementary Fig. 10).

## Discussion

Rodent models are the primary platforms for investigating human skin-associated infections, injuries, and diseases^10, 43^. Although rodent models provide insights into the mechanisms of human diseases and host response/defense against skin-associated infectious agents, several limitations exist in rodent models^10, 43^. It is well established that the human skin exhibits significant structural differences compared to rodent skin^10, 43^. These structural differences could play a critical role in skin-microbes interactions and cutaneous immune signaling, resulting in significant differences in anti-microbial host defense and inflammatory response between human and rodent skin^1, 2^. These differences could also significantly impact the translation of mechanistic findings from traditional rodent models to humans.

In an attempt to address the species differences between traditional rodent models of human skin diseases and skin diseases in humans, we engrafted human skin, autologous lymphoid tissues, and autologous hematopoietic stem cells into immunodeficient rodents (SRG rats and NSG mice). The human skin engrafted into immunodeficient rodents developed a microanatomical structure that resembled adult human skin. Human keratinocytes and dermal fibroblasts, which facilitate the development of multi-layered epidermis and dermis, respectively, were present in the human skin of both the hSIS-humanized rats and mice. To establish that the human skin xenograft on immunodeficient rats is susceptible to human pathogens, we infected the engrafted human skin with CA-MRSA via intradermal inoculation. The human skin xenograft in the hSIS rat model supports CA-MRSA-infection. The human skin in the hSIS-humanized mouse model also supports CA-MRSA-infection, albeit the infection appears to induce rapid death within three days. Prince et al. reported that human neutrophils and monocyte-derived macrophages are a target for CA-MRSA-associated Panton-Valentine leucocidin (PVL) in humanized mice with human immune cells and thymus xenograft ^48^; thus, enhanced inflammation could be mediating the rapid deaths. In the hSIS-humanized NSG mouse model, we observed readily detectable levels of human monocytes/macrophages in the blood and human skin. Future studies will investigate the interactions between human immune cell perturbations and CA-MRSA in the infected skin. A limitation of the hSIS-humanized mouse model is the short experimental window (< 2.5 months) for infection, as drying of the human skin xenograft (resulting in hardening) and early signs of murine hair loss (suggestive of graft-versus-host disease) are evident at approximately five months post-transplantation. hSIS-humanized SRG rats do not exhibit signs of graft versus host disease, even at nine months post-transplantation; thus, hSIS-humanized SRG rats could provide an in vivo model for studies requiring a wider experimental window (> 3 months).

Diverse leukocyte populations mediate human immune response; the hSIS-humanized mouse model supports the development of both innate and adaptive immune cells. The peripheral blood of hSIS-humanized mice is reconstituted with lymphocytes, granulocytes, monocytes, dendritic cells, and natural killer cells. Additionally, the hSIS-humanized mouse model supports the development of human thymus and spleen tissues. The presence of these human primary and secondary lymphoid tissues, coupled with the development of innate and adaptive immune cells, may allow this model to recapitulate de novo antigen-specific human immune responses to a skin infection. Interestingly, the hSIS-humanized rat model exhibited marginal human immune cell reconstitution in the peripheral blood, despite evidence of human immune cell reconstitution in the human skin and thymus, as well as the rat spleen. A transgenic variant of the SRG rat model, carrying human signal regulatory protein alpha, a negative regulator of macrophage phagocytosis, could facilitate human immune cell reconstitution in the blood^49, 50^.

In summary, we report the development of humanized NSG mouse and SRG rat models that incorporate human lymphoid tissue(s) and autologous full-thickness human skin with cutaneous immune cells. hSIS-humanized NSG mice and SRG rats could provide a means for studying skin infections^25, 48, 51–60^.

## Methods

### Construction of human Skin and Immune System-humanized rodents

Adult (8–10 weeks) male and female severely immunodeficient rodents, namely Non-obese Diabetic (NOD) strain mice (Jackson Laboratory, Stock No: 005557) and Sprague Dawley (SD) strain rats (Hera Biolabs), carrying mutations in interleukin-2 receptor subunit gamma (IL2Rγ), as well as in the Protein Kinase, DNA-Activated, Catalytic Subunit (PRKDC) (mice only) or recombination activating gene 2 (RAG2) (rat only) were obtained from vendor and bred in the Division of Laboratory Animal Resources (DLAR) facility at the University of Pittsburgh. Human fetal tissues were obtained from the Health Sciences Tissue Bank at the University of Pittsburgh. Human fetal tissues for constructing humanized rodents were handled and processed under biosafety level 2 conditions. Male and female rodents were myoablated via gamma radiation using cesium-137 irradiator, with mice receiving a dose of 150 rads and rats receiving a dose of 500 rads. Myoablated male and female mice were transplanted with human fetal-thymus, liver, and spleen in the kidney capsule, autologous CD34^+^ hematopoietic stem cells (via retroorbital injection of 0.2 × 10^6^ cells)^17^, and full-thickness human fetal skin on the panniculus carnosus of the mouse skin-excised dorsum^61–63^. Myoablated male and female rats were transplanted with human fetal-thymus and liver in the renal capsule, autologous CD34^+^ hematopoietic stem cells (via retroorbital injection of 0.5 × 10^6^ cells), and autologous full-thickness human fetal skin (less than four days old) on the panniculus carnosus of the rat-skin excised dorsum^61–63^. In some instances, rodents were only transplanted with full-thickness human fetal skin. Rodents were housed under specific-pathogen-free conditions and fed irradiated chow and autoclaved water.

### Immune cell reconstitution and functional assays

For evaluating human immune cell reconstitution at indicated time points, peripheral blood was collected from animals, and leukocytes were analyzed using flow cytometry^17^. Briefly, peripheral blood was collected from rodents and mixed with 20 mM Ethylenediaminetetraacetic acid (EDTA) at a 1:1 ratio, and single-cell leukocytes were prepared via red blood cells lysis using Ammonium-Chloride-Potassium (ACK) buffer. For evaluating human immune reconstitution in the human skin xenograft in the hSIS-humanized mouse model, human skin tissue was excised from an hSIS-humanized mouse. Skin samples were cut into small and digested with collagenase. The dermis was separated from the epidermis, and a single-cell suspension of dermal tissue was created using a gentleMACS dissociator (Miltenyi Biotech). Epidermal tissues underwent trypsinization in order to obtain a single-cell suspension. Single-cell suspensions were stained with a LIVE/DEAD Fixable Aqua Dead Cell Stain Kit (ThermoFisher Scientific) and fluorochrome-conjugated antibodies (anti-mouse CD45-BioLegend Cat. No. 103126, anti-human CD45-BioLegend Cat. No. 304014), fixed with formalin, and analyzed on a BD LSRFortessa™ cell analyzer—flow cytometer (BD Biosciences). Data were analyzed using FlowJo software (Dako). Leukocytes were selected based on forward, and side scatter measurements. Single-cell and live leukocytes were selected for further analysis of the percentage of human leukocytes (anti-human CD45^+^, hCD3^+^, hCD4^+^, CD8^+^, hγδTCR^+^, hCD19^+^, hCD14^+^, hCD16^+^, hCD66b^+^) and mouse leukocytes (anti-mouse CD45^+^). The analysis of the various human immune cell populations and subsets were gated on human leukocytes. Human T cells were also isolated from the thymus tissue in humanized SRG rats via immunomagnetic selection using an anti-human CD3 antibody (EasySep™ Human CD3 Positive Selection, Catalog # 17951, Stemcell Technologies) and treated without (vehicle) or with Gibco™ Dynabeads™ Human T-Activator CD3/CD28 (Cat. No. 111.61D, ThermoFisher Scientific) in the presence of recombinant IL2 and BD GolgiPlug (BD Biosciences) for 12 h. Human cytokine expression (hTNFα and hIFNγ) in human T cells were analyzed using BD LSRFortessa™ cell analyzer—flow cytometer (BD Biosciences), and the data were analyzed using FlowJo software (Dako).

### Gross/In situ immune cell analysis

Gross analysis of tissues was performed using a camera (8 megapixels), with animals either euthanized or anesthetized prior to photographing. Indicated tissue samples from humanized rodents or humans (adult human skin from a 77-year-old male, BioChain, catalog number: T2234218 or adult female breast skin, University of Pittsburgh Biospecimen Repository), were fixed with formalin and, subsequently, embedded in paraffin. Paraffin-embedded, fixed sections were stained via hematoxylin and eosin, or via indicated human antibodies^17^ (anti-human CD45-Biocare Medical Cat. No. CME PM016AA; anti-human CD3-Biocare Medical Cat. No. CME 324 A, B, C; anti-human CD68-Biocare Medical catalog number CM 033 A, B, C; anti-human CD20-Biocare Medical catalog number ACR 3004 A, B; anti-human alpha-smooth muscle actin; anti-pan cytokeratin, Clone AE1/AE3, Biocare Medical catalog number SKU: 011; anti-human fibroblast, Clone TE7, Millipore Sigma catalog number CBL271; anti-human CD207, Dendritics catalog number: DDX0362). The immunoreactivity of the antibodies was determined via incubation with DAB substrate (MACH 2 Detection Kit, Biocare Medical) and counterstaining with hematoxylin.

### CA-MRSA infection in the human skin in immunodeficient rodents

The human skin xenografts on the immunodeficient rodents (SRG rats and NSG mice) were inoculated with CA-MRSA USA300^64^ via intradermal injection with 1 × 10^8^ CFU for rats and 1 × 10^6^ CFU for mice; non-transplanted rodents were inoculated via subqutaneous injection at the same dosage. After three days, portions of equal weight human or rodent skin was excised, and bacterial load was determined based on the number of colony-forming units (CFU); CA-MRSA bacterial strain was confirmed via polymerase chain reaction (PCR).

### Ethical approval

De-identified human fetal tissues at the gestational age of 18 to 20 weeks were obtained from medically or elective indicated termination of pregnancy through Magee-Womens Hospital of the University of Pittsburgh Medical Center (UPMC), with the University of Pittsburgh, Health Sciences Tissue Bank. Written informed consent of the maternal donors was obtained in all cases, under a protocol reviewed and approved by the Institutional Review Board (IRB) of the University of Pittsburgh; approved guidelines and federal/state regulations were adhered to for all procedures. The use of de-identified human fetal tissues to construct humanized rodents was reviewed and approved by the University of Pittsburgh IRB Office. The use of de-identified human fetal tissues did not constitute human subjects research as defined under federal regulations [45 CFR 46.102(d or f) and 21 CFR 56.102(c), (e), and (l)]. The use of human fetal liver-derived hematopoietic stem cells was reviewed and approved by the Human Stem Cell Research Oversight (hSCRO) at the University of Pittsburgh. The use of a biological agent (CA-MRSA), recombinant DNA, and transgenic animals were reviewed and approved by the Institutional Biosafety Committee (IBC) at the University of Pittsburgh. All animal studies/experimental protocols were reviewed and approved by the Institutional Animal Care and Use Committee at the University of Pittsburgh and were conducted following approved guidelines, which adheres to the NIH guidelines for housing and care of laboratory animals.

## Supplementary information

Supplementary Information 1.

## Data Availability

The datasets generated during and/or analyzed by the authors during this study are available from the corresponding author on reasonable request.
