# Author Correction: Development of humanized mouse and rat models with full-thickness human skin and autologous immune cells

**DOI:** 10.1038/s41598-021-98417-7

**Published:** 2021-09-14

**Authors:** Yash Agarwal, Cole Beatty, Sara Ho, Lance Thurlow, Antu Das, Samantha Kelly, Isabella Castronova, Rajeev Salunke, Shivkumar Biradar, Tseten Yeshi, Anthony Richardson, Moses Bility

**Affiliations:** 1 Department of Infectious Diseases and Microbiology, University of Pittsburgh, Pittsburgh, USA; 2 Department of Microbiology and Molecular Genetics, University of Pittsburgh, Pittsburgh, USA; 3 Hera Biolabs, Inc, Lexington, USA

Correction to: *Scientific Reports* 10.1038/s41598-020-71548-z, published online 03 September 2020

The Acknowledgements section in the original version of this Article was incorrect.

“We used the UPMC-Hillman Cancer Center and Tissue and Research Pathology within the University of Pittsburgh Biospecimen Core, which is supported in part by the NIH award P30CA047904. Miss Vanshika Narala and Miss Nivitha Periyapatna, Department of Infectious Diseases and Microbiology, University of Pittsburgh assisted with collecting histological data and analysis. Berthony Deslouches, Department of Microbiology and Molecular Genetics, University of Pittsburgh provided insightful advice on developing this project. This work was supported by the National Institutes of Health (NIH)-National Institute of Allergy and Infectious Diseases (NIAID) (R21AI135412) and National Institutes of Health (NIH)-Fogarty International Center (FIC) (D43TW010039). The National Institute of Health, which funds this work, requires scientists to submit final peer-reviewed journal manuscripts that arise from NIH funds to the digital archive PubMed Central upon acceptance for publication.”

now reads:

“We used the UPMC-Hillman Cancer Center and Tissue and Research Pathology within the University of Pittsburgh Biospecimen Core, which is supported in part by the NIH award P30CA047904. Miss Vanshika Narala and Miss Nivitha Periyapatna, Department of Infectious Diseases and Microbiology, University of Pittsburgh assisted with collecting histological data and analysis. Berthony Deslouches, Department of Microbiology and Molecular Genetics, University of Pittsburgh provided insightful advice on developing this project. This work was supported by the National Institutes of Health (NIH)-National Institute of Allergy and Infectious Diseases (NIAID) (R21AI135412). The National Institute of Health, which funds this work, requires scientists to submit final peer-reviewed journal manuscripts that arise from NIH funds to the digital archive PubMed Central upon acceptance for publication.”

The original Article has been corrected.

