# Supplementary material for: Development of humanized mouse and rat models with full-thickness human skin and autologous immune cells
Source: Sci Rep. 2020 Sep 3;10:14598. doi: 10.1038/s41598-020-71548-z (PMC7471691; doi:10.1038/s41598-020-71548-z)
Supplement: Supplementary file 1 — Supplementary Information 1. [file 41598_2020_71548_MOESM1_ESM.docx]

**Development of humanized mouse and rat models with full-thickness human skin and autologous immune cells**

Authors: Yash Agarwal^1#^, Cole Beatty^1#^, Sara Ho^1#^, Lance Thurlow^2^, Antu Das^1^, Samantha Kelly^1^, Isabella Castronova^1^, Rajeev Salunke^1^, Shivkumar Biradar^1^, Tseten Yeshi^3^, Anthony Richardson^2^, and Moses Bility^1*^.

Affiliations: ^1^Department of Infectious Diseases and Microbiology and ^2^Department of Microbiology and Molecular Genetics, University of Pittsburgh; ^3^Hera Biolabs, Inc.

**^#^**denotes the authors contributed equally to the work**; ^*^**denotes the corresponding author.

**Supplementary Figures and Legends**

**Supplementary Figure 1. Development of** **human skin and Immune System-humanized NSG mouse model.** Transplantation of full-thickness human skin, autologous hematopoietic stem cells, and lymphoid tissue (spleen and thymus) pieces in immunodeficient NSG mice results in the development of human skin and Immune System-humanized NSG mice, and a success rate, as defined by maintaining engrafted human cells and tissues for >10 weeks, of over 75%. These long-term experiments involved three independent cohorts, with 6-10 mice per cohort, totaling 26 mice.

**
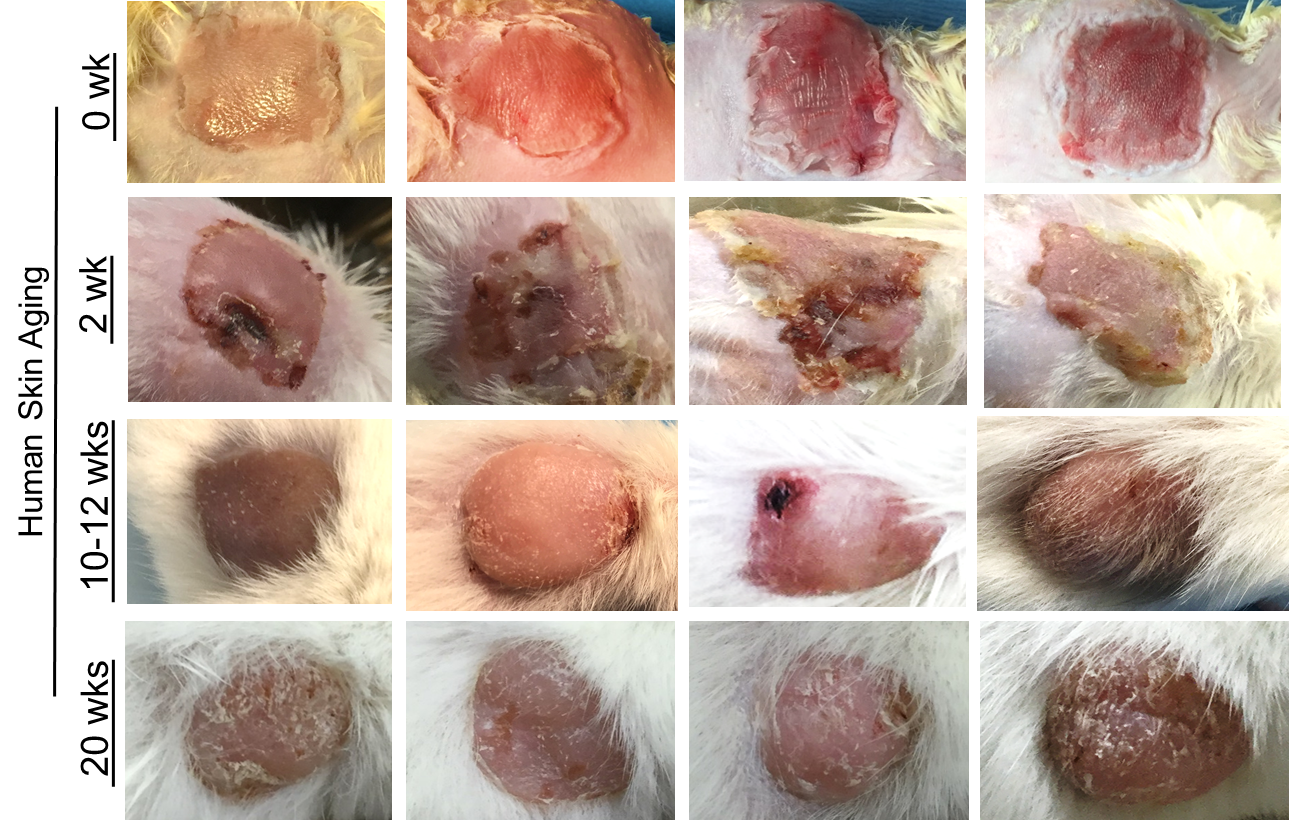
**

**Supplementary Figure 2. Human skin development in the human Skin and Immune System-humanized NSG mouse model.** Transplantation of full-thickness human skin on the dorsum of hSIS-mice results in the development of full-thickness human skin as exhibited in representative gross-photos at 0- (the day of transplantation), 2-, 10-, 12- and 20-weeks post-transplantation (n=4 per group); drying of the human skin graft and murine hair loss emerges at 20-weeks post-transplantation.


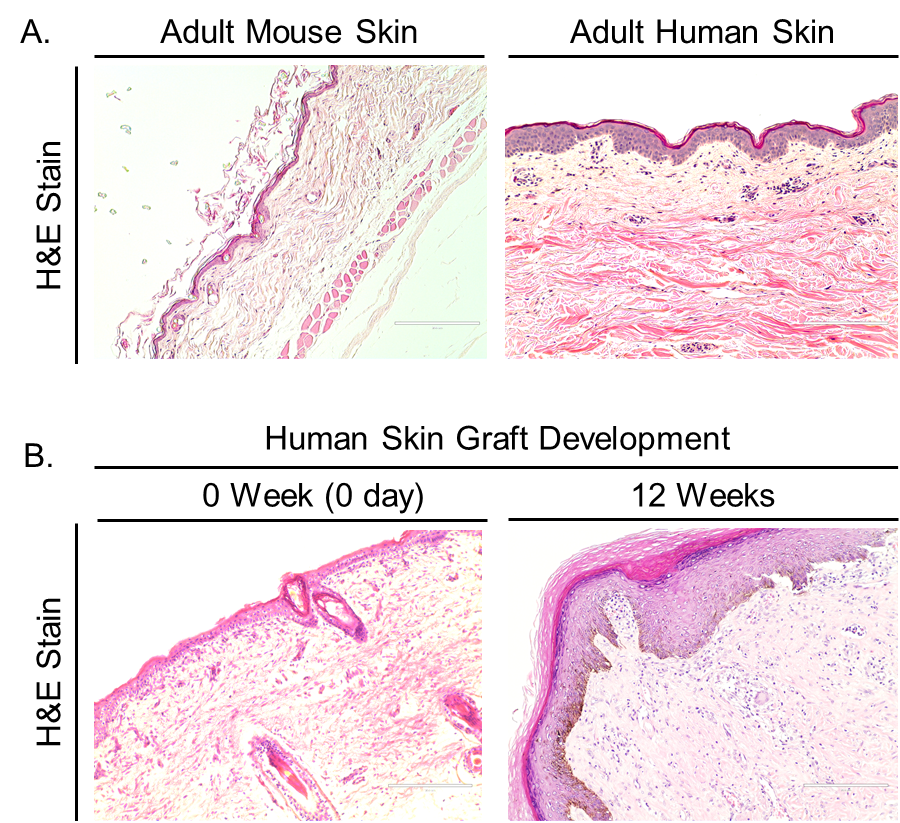


**Supplementary Figure 3. Comparative analysis of human and mouse skin tissues in respective hosts and the** **human Skin and Immune System-humanized NSG mouse model.** Representative histological (H&E) analysis of healthy adult-mouse skin (6 months old NSG mice) (n=4) and healthy adult-human skin (77-year-old male) (n=2) (A) demonstrates the well-established difference between the species, namely, the absence of multicellular layer (>5 layers) epidermis in mice. (B) Representative histological (H&E) analysis of the human fetal skin before transplantation (n=4) and the engrafted human fetal skin at 12 weeks post-transplantation (n=4) in human Skin and Immune System-humanized NSG mice demonstrate the development of the dermis, multicellular layer (>5 layers) epidermis and cornified envelope. The human fetal skin before transplantation has a relatively thin epidermis, underdeveloped dermis, and lacks a cornified envelope. Scale bars: 200 μm.


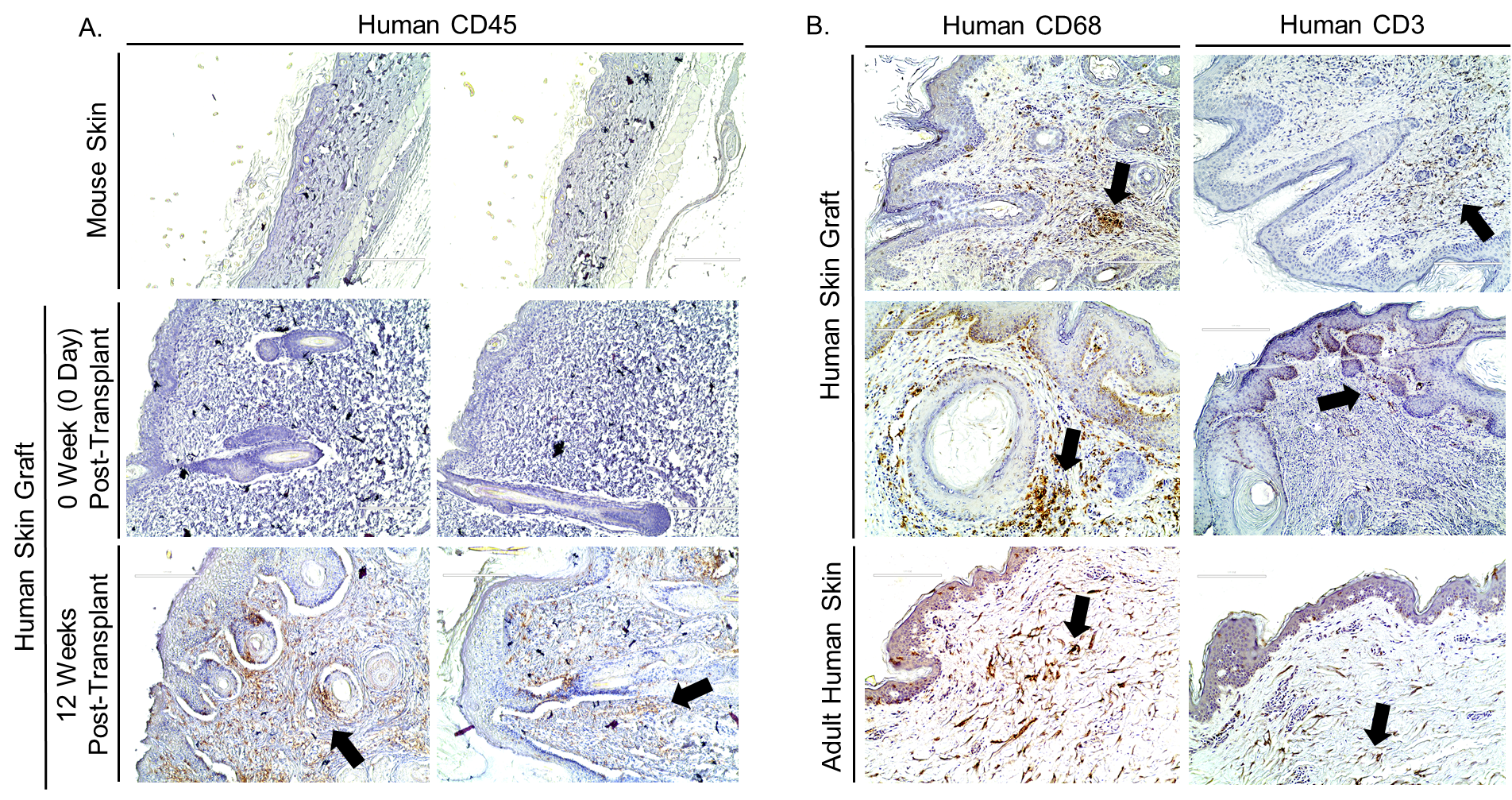


**Supplementary Figure 4. Development of human cutaneous immune cells** **in the human Skin and Immune System-humanized NSG mouse model.** (A) Representative immunohistochemical analysis of the human skin in human Skin and Immune System-humanized NSG mice demonstrate the presence of human cutaneous immune cells (hCD45+ cells) at 12-weeks post-transplantation (n=3), with those cells undetectable in the human fetal skin (n=3) before transplantation. Human CD45+ cells are also undetectable in non-transplanted mouse skin, which serves as a staining control (n=3). (B) Representative immunohistochemical analysis of human cutaneous immune cell subpopulations in human Skin and Immune System-humanized NSG mice demonstrate the presence of human macrophages (hCD68+ cells) and T cells (hCD3+) at 12-weeks post-transplantation (n=3), at levels comparable to healthy adult-human skin (n=2). Scale bars: 200 μm.

**
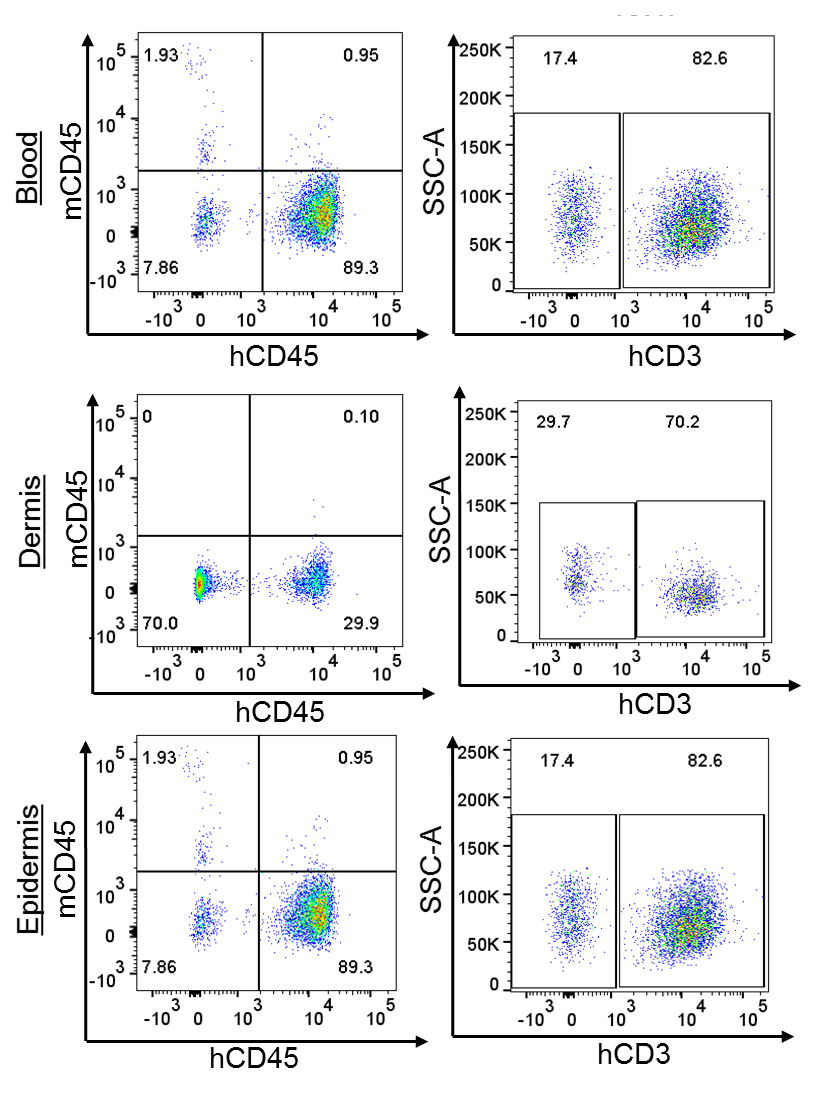
Supplementary Figure 5. Development of peripheral and cutaneous human immune cells in the human Skin and Immune System-humanized NSG mouse model.** Flow cytometric analysis of human immune cell reconstitution in the peripheral blood and the human skin xenograft (dermis and epidermis) in a human Skin and Immune System (hSIS)-humanized mouse at five months post-transplantation demonstrates the presence of human immune cells (hCD45+ cells) (Left Panel), with human T cells (hCD45+, hCD3+ cells) making up a significant portion of those human immune cells (Right Panel). Negligible levels of mouse immune cells (mCD45+) were present in the blood and the human skin.

**
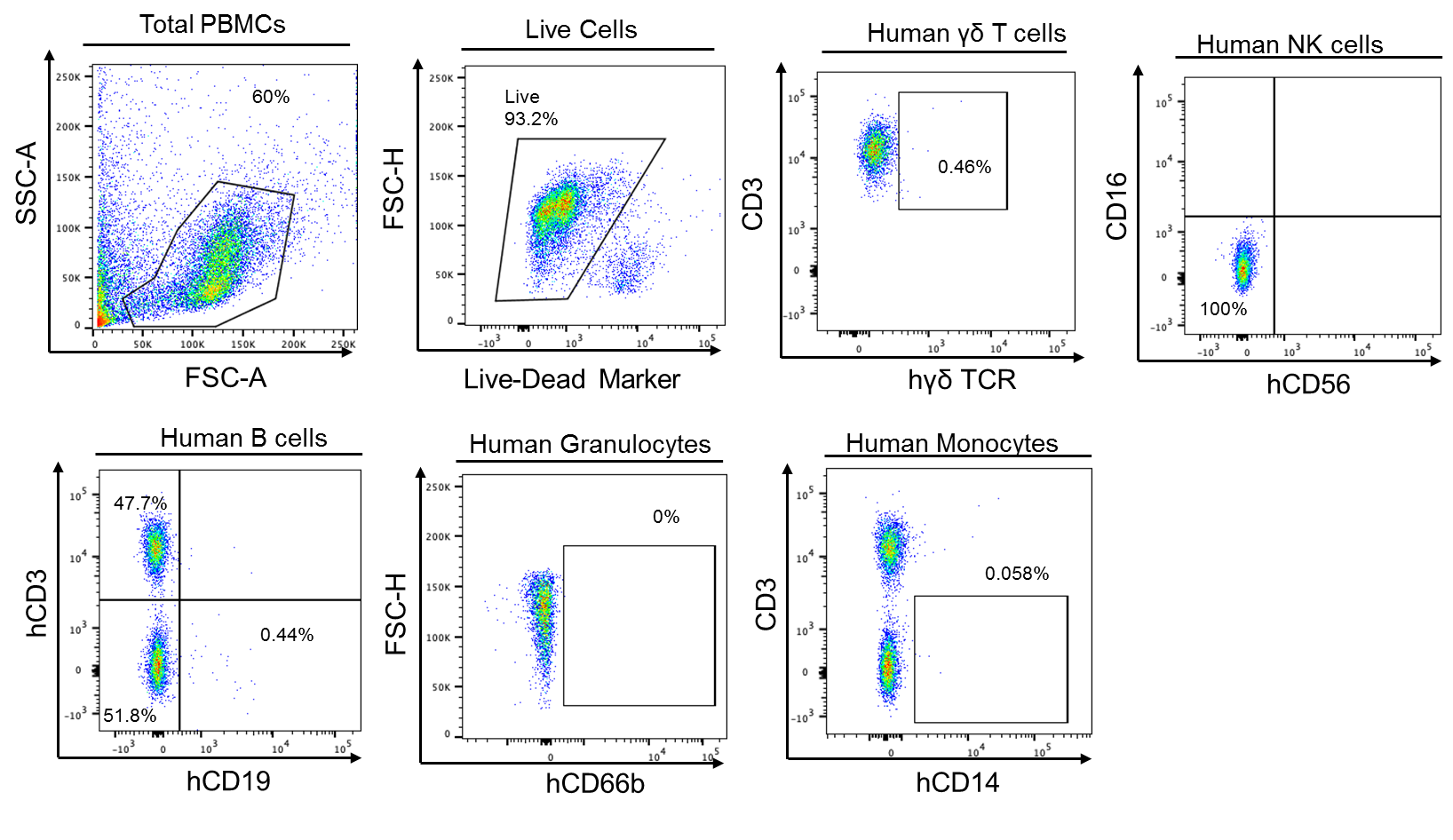
Supplementary Figure 6. Flow cytometry controls for identifying human immune cell subpopulations in the peripheral blood in the** **human Skin and Immune System-humanized NSG mouse model.** Representative flow cytometry analysis of isotype control stains of peripheral blood mononuclear cells (PBMCs) from human Skin and Immune System-humanized NSG mice at 10-12 weeks post-transplantation demonstrates appropriate negative controls for the gating of live human PBMC (hCD45+) subtypes (B cells-hCD19+ hCD3- human PBMCs, αβ T cells-hCD3+ human PBMCs, hCD4+ T cells, hCD8+ T cells, hγδTCR+ T cells- hγδ TCR+ CD3+ human PBMCs, natural killer cells (NK)-hCD57+ hCD3- human PBMCs, monocytes (hMo)-hCD14+ CD3- human PBMCs, and granulocytes (hPMN)-hCD66b+ hCD3- human PBMCs.

**Supplementary Figure 7. Development of human Skin and Immune System-humanized SRG rat model.** Transplantation of full-thickness human skin, autologous hematopoietic stem cells, and lymphoid tissue (thymus) pieces in immunodeficient SRG rat results in the development of the human Skin and Immune System-humanized SRG rat model, and a success rate of 100% in maintaining those engrafted human tissues for >6 months. These experiments were conducted with 3 independent cohorts, with 5-7 mice per cohort, totaling 18 rats.

**
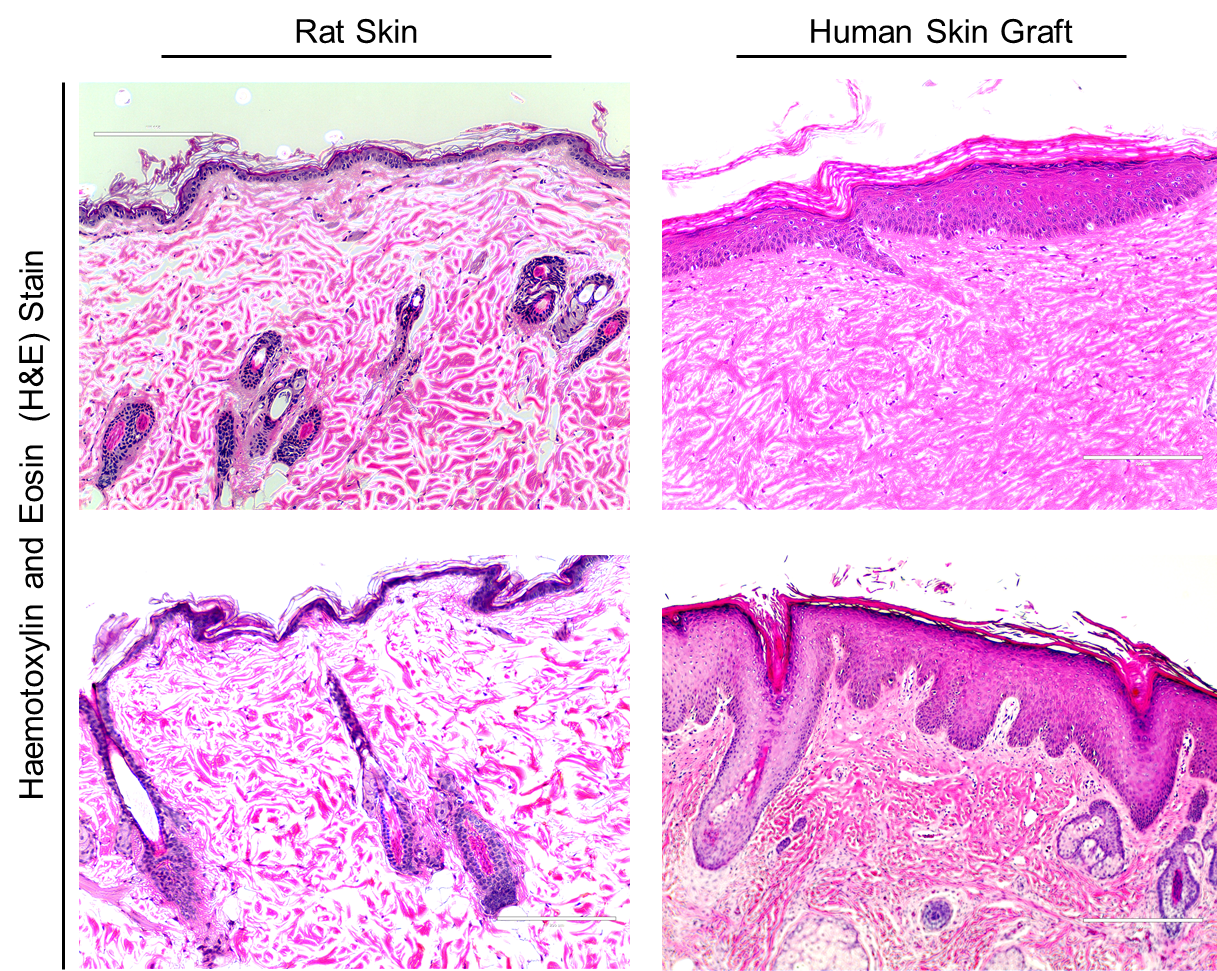
**

**Supplementary Figure 8. Comparative analysis of SRG rat skin and the human skin xenograft in the human Skin and Immune System-humanized SRG rat model.** Representative histological (H&E) analysis of healthy adult-rat skin (8 months old SRG rats) (n=2) and human skin xenograft in human Skin and Immune System-humanized SRG rats (n=4) demonstrates the well-established difference between the species, namely, the absence of multicellular layer (>5 layers) epidermis in rats. Scale bars: 200 μm.

**
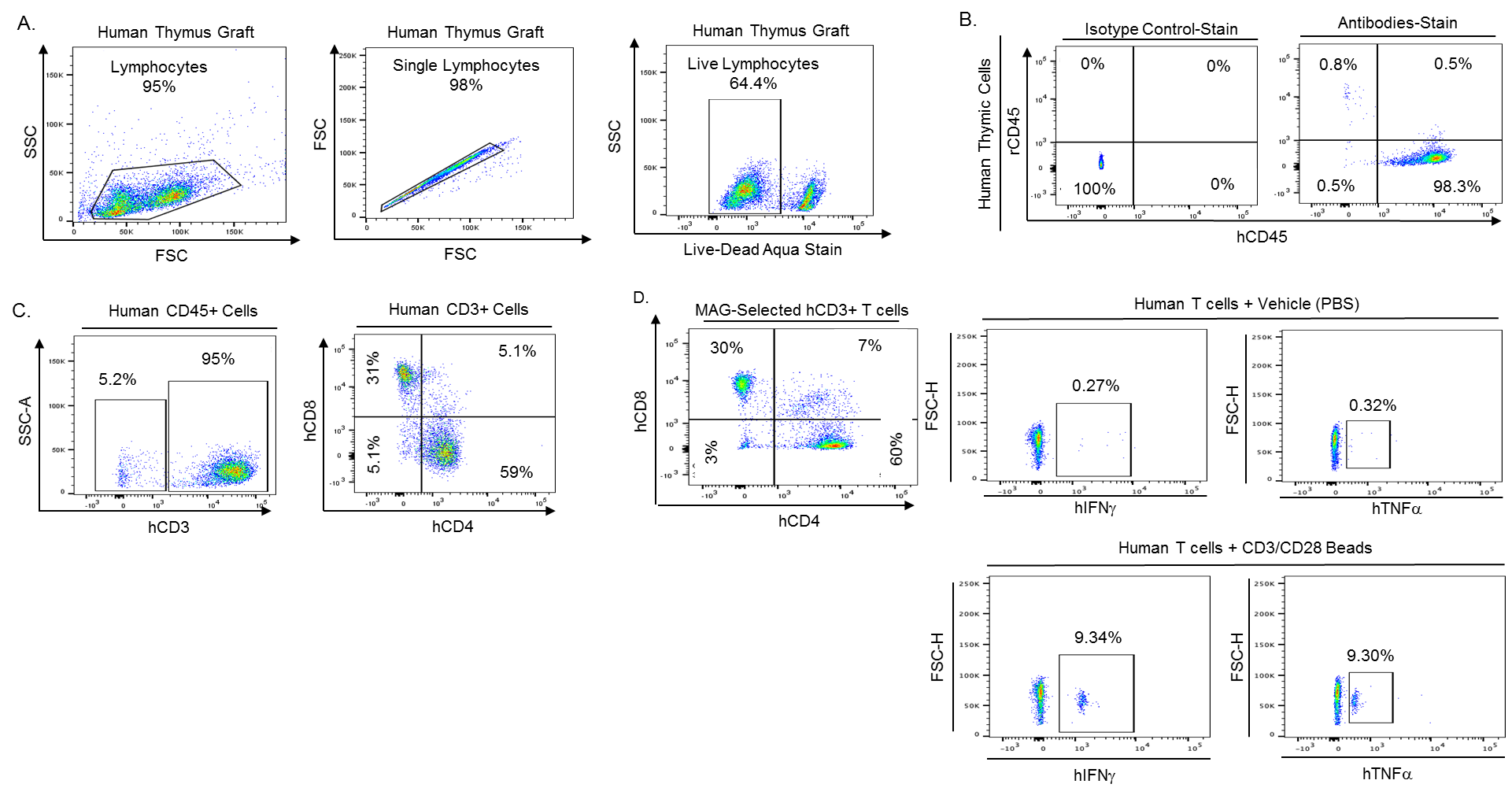
**

**Supplementary Figure 9. Human Skin and Immune System-humanized SRG rat model supports the development of functional human T cells.** (A-C) Flow cytometry analysis of the human thymus xenograft in the human Skin and Immune System-humanized SRG rat model at 36-weeks post-transplantation, demonstrate the presence of human immune cells (hCD45+) (A-B), with high levels of human T cells (hCD3+) (including human CD4+ and CD8+ T cells) and marginal levels of human CD3- cells (hCD3-) (C). (D) Flow cytometry analysis of human T cell (hCD3+) selection from human thymic cells in the human Skin and Immune System-humanized SRG rat model at 36-weeks post-transplantation using human CD3+ immunomagnetic beads demonstrates the isolation of human T cells (including human CD4+ and CD8+ T cells). Flow cytometry analysis of human Th1 cytokines (human INFγ and TNFα) in those isolated human T cells (hCD3+) following treatment without (vehicle) or with human CD3/CD28 beads demonstrates elevated levels of human Th1 cytokines in response to stimulation.

**Supplementary Figure 10. Human skin xenograft in hSIS-humanized mouse model support CA-MRSA colonization.** Community-associated *methicillin-resistant Staphylococcus aureus* (CA-MRSA) was inoculated (intradermal injection) into the engrafted human skin in hSIS-humanized NSG mouse (Humanized) at ten weeks post-transplantation. Additionally, CA-MRSA was inoculated (subqutaneous injection) into the mouse skin of non-transplanted NSG mouse (Non-transplanted) for comparative purposes. Bacterial load in the inoculated skin tissues at three days post-inoculation as measured via colony-forming unit (CFU) assay demostrate skin infection. Two additional hSIS-humanized mice at 10-12 weeks post-transplantation, died within three days following intradermal CA-MRSA inoculation.
